# Provisional Tic Disorder is not so transient

**DOI:** 10.1038/s41598-019-40133-4

**Published:** 2019-03-08

**Authors:** Soyoung Kim, Deanna J. Greene, Emily C. Bihun, Jonathan M. Koller, Jacqueline M. Hampton, Haley Acevedo, Angela M. Reiersen, Bradley L. Schlaggar, Kevin J. Black

**Affiliations:** 1 Department of Psychiatry, Washington University School of Medicine, St. Louis, MO, United States; 2 Department of Radiology, Washington University School of Medicine, St. Louis, MO, United States; 3 Department of Neurology, Washington University School of Medicine, St. Louis, MO, United States; 4 Kennedy Krieger Institute, Baltimore, MD United States; 5 Department of Neurology, Johns Hopkins University School of Medicine, Baltimore, MD United States; 6 Department of Pediatrics, Johns Hopkins University School of Medicine, Baltimore, MD United States; 7 Department of Neuroscience, Washington University School of Medicine, St. Louis, MO, United States

## Abstract

Motor and vocal tics are common in childhood. The received wisdom among clinicians is that for most children the tics are temporary, disappearing within a few months. However, that common clinical teaching is based largely on biased and incomplete data. The present study was designed to prospectively assess outcome of children with what the current nomenclature calls Provisional Tic Disorder. We identified 43 children with recent onset tics (mean 3.3 months since tic onset) and re-examined 39 of them on the 12-month anniversary of their first tic. Tic symptoms improved on a group level at the 12-month follow-up, and only two children had more than minimal impairment due to tics. Remarkably, however, tics were present in all children at follow-up, although in several cases tics were apparent only when the child was observed remotely by video. Our results suggest that remission of Provisional Tic Disorder is the exception rather than the rule. We also identified several clinical features present at the first examination that predict one-year outcome; these include baseline tic severity, subsyndromal autism spectrum symptoms, and the presence of an anxiety disorder.

## Introduction

Tics are sudden, rapid, recurrent, nonrhythmic movements or vocalizations such as eye blinking or throat clearing^1,2^. Tics are common in childhood, with a generally accepted prevalence rate of 20%^3–6^, though we have argued that the true prevalence may be much higher^7^. On the other hand, only about 3% of all children have tics for the year that is required to diagnose Tourette syndrome or a chronic tic disorder (hereafter “TS”)^3,8^. When tics have not yet lasted a year since onset, Provisional Tic Disorder (PTD) is diagnosed^2^. Since every child with chronic tics could have been diagnosed with PTD during the first year after tics started, some children with PTD must experience marked improvement or remission of tics within the first year, while others go on to a diagnosis of TS. Thus, TS can be conceptualized as a two-step process: tics appear, and then they fail to remit. The epidemiological data summarized above would suggest that the second step is the more unusual, with PTD remitting the large majority of the time. However, little existing research on tics has focused on PTD.

Nevertheless, studying the PTD population is important, both in its own right and for the implications for TS. Children with recent-onset tics and their parents want to know prognosis. At present, there is almost no information to determine which children will go on to need long-term treatment. Studying this population also offers the tantalizing possibility of secondary prevention of tic disorders (*i.e*., preventing progression to chronicity). Previous cross-sectional studies that find an association between TS and a particular biological marker usually are unable to determine whether the association is causal, and if so whether the marker is a consequence or cause of the disease^9^. By contrast, a prospective study, starting before tics become chronic, may answer these questions. For example, abnormalities in TS that are also present in PTD are unlikely to reflect chronicity or adaptation. Additionally, the causes of tic appearance and of tic disappearance may differ. Unfortunately, few studies have targeted patients during the first year of ticcing and their findings are generally retrospective, sparse, and contradicting^10–12^. While limited prospective data suggest that only a third of children with PTD show complete remission of tics over the next 5–10 years^7^, the epidemiology reviewed above suggests otherwise, and experts have opined that PTD usually remits within a few months^2,10,13–21^.

Here we present the largest face-to-face, prospective study of clinical outcomes in PTD. This project includes structural and functional MRI, a standardized tic suppression protocol, and various neuropsychological tests (www.NewTics.org). Here we report the clinical outcomes of the first 43 participants at 12 months after the first tic (*i.e*., when TS can first be diagnosed). We also explored baseline clinical predictors (collected at the first visit) of tic outcome at 12 months. The study continues, but the protocol changed after these 43 were enrolled; thus, this cohort is well suited for investigation of the clinical data.

## Results

### Baseline characteristics

Table 1 summarizes the clinical features of participants at baseline and at the 12-month follow-up visit. Their baseline visit occurred on average 3 months after their first tic, at a mean age of 8.1 years (all but 4 were 5.0–10.9 years old). Past week tic severity at the baseline visit was modest on average, with a mean YGTSS score (total tic score [TTS] + impairment score) of 24.98. The TTS ranged from 7 to 30; 5 participants showed minimal tic severity (TTS ≤ 10), 29 showed mild tic severity (score 11–20), and 9 showed moderate to severe tic severity (score > 20). The impairment score ranged from 0 to 30; 33 participants reported minimal impairment (score 0–10), 7 reported mild impairment (11–20), and 3 reported moderate impairment (score 21–30). Only 7 out of 35 participants were judged (by KJB) to ever have had impairment in a life role or marked distress associated with tics (not recorded for 8 participants). As expected, participants had experienced fewer classic TS symptoms during the short course of their illness than a typical TS population (mean DCI score at baseline was 31.84 ± 13.95; though the range was broad: 14–80). Nevertheless, the symptoms were generally concerning to parents; the parents of 12 out of 15 participants answered “yes” when asked at screening if they would be taking their child to a doctor because of the tics (this question was added midway through the study). Nearly all participants met criteria for another psychiatric diagnosis. Specifically, K–SADS revealed one or more diagnosis other than tic disorders in 35 participants: 18 participants met criteria for ADHD, 6 participants had OCD, and 20 participants had an anxiety disorder (other than OCD).

**Table 1:** Characteristics of study participants at the baseline and 12-month follow-up visit.

Descriptor	Baseline All participants n = 43	Baseline 12-month completed n = 39	12-month Follow-up n = 39	t	p
	mean ± sd or count	mean ± sd or count	mean ± sd or count		
Age	8.13 ± 2.43	7.97 ± 2.28			
Sex	30 M/13F	26 M/13F			
Handedness	39 R/4L	35 R/4L			
Non-white	4	3			
Barratt SES	50.81 ± 10.76	50.99 ± 10.96			
K-BIT IQ	109.23 ± 12.83	109.28 ± 12.5			
Days since tic onset	99.74 ± 41.91	98.38 ± 43.39	372.79 ± 15.43		
YGTSS Total	24.98 ± 12.57	24.85 ± 12.8	18.13 ± 11.4	2.86	0.01
- TTS (total tic)	17.23 ± 6.15	17.21 ± 6.13	14.15 ± 6.63	2.86	0.01
- Motor	10.95 ± 3.71	10.87 ± 3.83	9.15 ± 4.19	2.8	0.01
- Phonic	6.28 ± 4.58	6.33 ± 4.55	5 ± 3.54	1.76	0.09
- Impairment	7.74 ± 7.81	7.64 ± 8.09	3.85 ± 5.79	2.42	0.02
- Minimal Impairment	33	30	37		
- Complex Tics (Present)	30	27	21		
Vocal Tics (ever)	35	32	35		
PUTS*	13.03 ± 4.57	12.65 ± 3.84	15.67 ± 6.11	−2.7	0.01
DCI	31.84 ± 13.95	30.87 ± 13.7	41.28 ± 16.31	−7.2	0
1st degree relative with tics	16	15			
- Parent with tics	10	9			
Non-tic K-SADS Diagnosis	35	31			
ADHD Diagnosis	18	14			
OCD Diagnosis	6	5			
Anxiety Disorder	20	20			
Medication	5	3			
PedsQL - Total	84.37 ± 10.34	83.84 ± 10.63	85.34 ± 11.53	−0.9	0.39
- Physical Functioniing	90.62 ± 11.81	89.98 ± 12.17	90.87 ± 13.13	−0.4	0.7
- Emotional Functioning	79.3 ± 18.08	77.69 ± 18.2	80.9 ± 18.24	−1.4	0.17
- Social Functioning	86.63 ± 16.79	85.51 ± 17.2	90.9 ± 10.06	−2.3	0.03
- School Functioning	80.93 ± 16.08	82.18 ± 15.68	78.72 ± 17.69	1.31	0.2
SRS total T scores	49.79 ± 8.55	50.08 ± 8.62			
- Awareness T scores	53.19 ± 9.08	53.38 ± 9.07			
- Cognition T scores	49.7 ± 8.24	49.92 ± 8.33			
- Communication T scores	48.6 ± 8.52	48.87 ± 8.61			
- Motivation T scores	50.23 ± 9.36	50.41 ± 9.57			
- Mannerisms T scores	50.56 ± 10.25	50.95 ± 10.45			
CBCL – DESR**	165.63 ± 14.2	166.21 ± 14.6	164.95 ± 18.44	0.42	0.68
- Anxious/Depressed	54.56 ± 6.25	55 ± 6.41	54.89 ± 7.59	0.03	0.97
- Attention Problems	56.02 ± 6.14	55.92 ± 6.25	55.73 ± 8	−0.2	0.86
- Aggressive Behavior	55.05 ± 6.16	55.28 ± 6.38	54.32 ± 5.83	0.97	0.34

Continuous data are summarized as mean ± SD. Paired t tests were conducted to compare the baseline and 12-month follow-up visits where applicable. “Non-white” includes 1 participant who did not report race. “YGTSS – Minimal Impairment” indicates the number of participants who scored 10 or below on the YGTSS Impairment scale. “Anxiety Disorder” includes panic disorder, separation anxiety disorder, social anxiety disorder, agoraphobia, specific phobia, generalized anxiety disorder (DSM-IV), and avoidant disorder of childhood (DSM-III-R).

*37 participants completed the PUTS at baseline, 34 of whom completed the 12-month follow-up visit; 36 children completed the PUTS at the 12-month follow-up visit.

**All participants completed the CBCL at baseline, but 37 completed the CBCL at the 12-month follow-up visit.

### Outcome

Four children did not return for their 12-month visit. Clinical features of the group at baseline did not significantly change with the removal of these children, except that all four had ADHD (Table 1). Outcomes are reported for the remaining 39 children. Overall, tic severity improved over time. The YGTSS TTS declined by an average of 18% at the 12-month visit compared to the baseline visit (*p* = 0.007; Table 1). The number of participants with a current complex tic decreased from 27 to 21. All but two participants reported minimal impairment (YGTSS impairment ≤10) at the 12-month visit. Few parents (3 of 29 asked) were planning to seek further clinical care at this time for their child’s tics. Only six met DSM-IV criteria for Tourette’s Disorder (which require impairment in a life role or marked distress). In fact, at the 12-month visit, several (at least 9 of 28) had no tics observed during extensive clinical history review or neurological examination.

Remarkably, however, at follow-up all children had tics within the past week. Three children and their parents (out of 29) said they had not noticed any tic within the past week (not explicitly recorded for 10 early subjects). However, these three children showed tics when alone (observed remotely by video). Out of 39 participants, all but one child showed tics when observed alone; the child with no tics on video had parent-reported tics within the past week and was observed to tic on a later encounter. Thus, all but four children met DSM-IV-TR criteria (which do not require impairment or marked distress) for Tourette’s Disorder and three of those four met criteria for Chronic Motor Tic Disorder (See Table 2). The remaining child had reportedly experienced an intervening asymptomatic period of a few months, for a DSM-IV-TR diagnosis of a second episode of Transient Tic Disorder. Every child met DSM-5 criteria for Tourette’s Disorder or Chronic Motor Tic Disorder (as DSM-5 does not exclude brief remissions). Even if all 4 of the participants lost to follow-up remitted completely, at least 90% (39/43) of the total sample still showed tics at the one-year anniversary of tic onset. With 95% confidence, these numbers indicate that tics persist to the one-year mark in at least 78% of children with PTD [95% confidence interval (CI) for the proportion 39/43 = 0.784, 0.963^22^]. This percentage is much higher than previously accepted estimates.

**Table 2:** Participant diagnoses.

Study ID	Tic Disorder				Comorbidity		Doctor?	
	Baseline	12-month Follow-up			Baseline	12-month follow-up	Baseline	12-month follow-up
	DSM-5	DSM-IV	DSM-IV-TR	DSM-5				
NT701	PTD	ND	TS	TS	ADHD	ADHD	—	No
NT702	PTD	ND	TS	TS	OCD	OCD	—	—
NT703	PTD	ND	TS	TS	ADHD	ADHD	—	No
NT704	PTD	N/A	N/A	N/A	ADHD, OCD	N/A	—	—
NT705	CMTD	N/A	N/A	N/A	ADHD	N/A	—	—
NT707	PTD	TS	TS	TS	ADHD	ADHD	—	—
NT709	PTD	N/A	N/A	N/A	ADHD	N/A	—	—
NT710	PTD	ND	TS	TS	ADHD	ADHD, OCD	—	—
NT712	PTD	ND	TS	TS	ND	ND	—	—
NT713	PTD	ND	TS	TS	ADHD	ADHD	—	—
NT714	TS	ND	TS	TS	ND	ND	—	—
NT715	PTD	ND	TTD*	CMTD	OCD	ADHD	—	—
NT716	PTD	ND	TS	TS	ADHD, OCD	ND	—	—
NT718	PTD	ND	TS	TS	ND	ND	—	—
NT719	PTD	TS	TS	TS	ND	ADHD	—	—
NT720	PTD	TS	TS	TS	OCD	ADHD, OCD	—	—
NT722	PTD	ND	CMTD	CMTD	ADHD	ADHD	—	No
NT724	PTD	ND	TS	TS	ND	ND	—	No
NT726	PTD	TS	TS	TS	ADHD	ADHD, OCD	—	Yes
NT728	PTD	ND	TS	TS	ADHD, OCD	ADHD	—	No
NT729	PTD	ND	TS	TS	ND	ND	—	No
NT730	PTD	ND	TS	TS	ND	ND	—	No
NT731	PTD	ND	TS	TS	N	ND	—	No
NT732	PTD	ND	TS	TS	ND	OCD	—	No
NT733	PTD	ND	TS	TS	ND	ND	Yes	Yes
NT734	TS	ND	TS	TS	ND	ADHD	—	No
NT735	PTD	ND	TS	TS	ND	ND	Yes	No
NT736	PTD	TS	TS	TS	ADHD	ADHD, OCD	Yes	No
NT738	PTD	TS	TS	TS	ND	ND	Yes	No
NT801	PTD	ND	TTD*	TS	ND	ND	Yes	No
NT805	PTD	ND	TS	TS	ADHD	ND	No	No
NT806	PTD	ND	TS	TS	ND	ND	—	No
NT807	PTD	ND	TS	TS	ND	ND	Yes	No
NT808	PTD	ND	CMTD	CMTD	ND	ND	No	No
NT809	PTD	ND	TS	TS	ADHD	ADHD	Yes	No
NT810	PTD	ND	TS	TS	ND	ND	Yes	No
NT811	PTD	ND	TS	TS	ND	OCD	—	No
NT812	PTD	ND	TS	TS	ND	OCD	Yes	No
NT813	PTD	N/A	N/A	N/A	ADHD	N/A	Yes	Yes
NT814	PTD	ND	TS	TS	ADHD	ADHD	Yes	No
NT815	PTD	ND	TS	TS	ND	ND	Yes	Yes
NT816	PTD	ND	TS	TS	ND	ND	—	No
NT817	PTD	ND	TS	TS	ADHD	ADHD	No	No

In the DSM-IV column, ND indicates No Diagnosis; alternatively one might diagnose Tic Disorder Not Otherwise Specified (for which the criteria do not specify impairment or marked distress). TTD* in the DSM-IV-TR column indicates a second (or later) episode of TTD; tics were present at the 12-month follow-up visit in each of these cases. TS is used for Tourette’s Disorder. “Doctor?”: Parent answered “yes” to the question, [excluding the visit for this research study], “would you be taking your child to the doctor now (or in the near future) because of his/her tics?”. Dash (−) indicates missing data. N/A indicates a participant who did not complete the 12-month follow-up visit. CMTD: chronic motor tic disorder; PTD: provisional tic disorder; TS: Tourette syndrome; TTD: transient tic disorder. “ADHD” includes ADHD Not Otherwise Specified as well as any subtype of ADHD.

DCI scores, which measure typical features of TS, increased from the baseline to the 12-month visit. The DCI score cannot decrease, as it represents a cumulative account of features ever observed, but this change indicates that additional clinical features continued to accrue over the 6- to 12-month interval between visits. The mean DCI score at the 12-month visit was 41.28, representing a fairly typical history for early TS, but the range was broad (13–74). Pearson’s correlation analysis revealed a significant correlation between TTS and DCI scores at both the baseline visit, *r = *0.507, *p* < 0.001, and the 12-month visit, *r* = 0.676, *p* < 0.001. The change in these two variables between visits, ΔTTS and ΔDCI, also correlated, *r* = 0.373, *p* = 0.019, such that the participants who showed a smaller reduction (or a greater increase) in TTS at the 12-month visit also showed a greater increase in DCI at the 12-month visit.

PUTS scores, reflecting awareness of premonitory symptoms, also increased by the 12-month visit. There was no significant correlation between TTS and PUTS either at the baseline visit (*r*_*s*_ (35) = −0.07, *p* = 0.68) or the 12-month follow-up visit (*r*_*s*_ (34) = 0.26, *p* = 0.13) or between ΔTTS and ΔPUTS (*r*(30) = −0.02, *p* = 0.90). Previous studies have noted that the PUTS can be less reliable in children under 10 years old, as their ability to report premonitory urges can be limited^23,24^. Six participants at the baseline visit and three participants at the 12-month visit were not able to complete the PUTS due to the difficulty in reporting these internal phenomena.

### Prognosis

**Figure 1:**
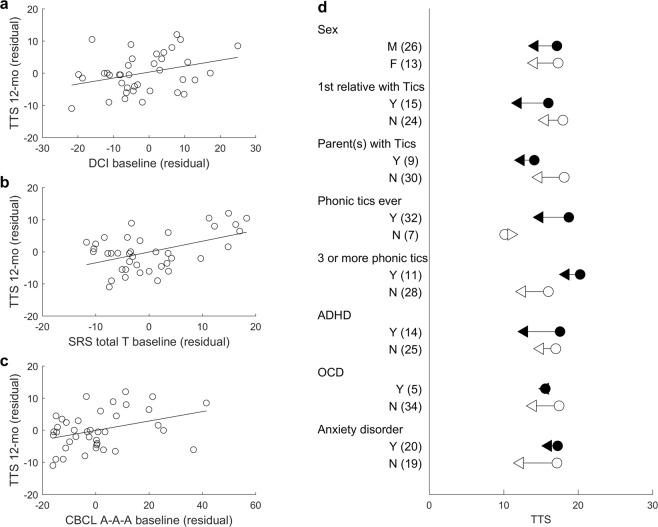
Predictors of tic severity outcome. Plots display the correlations between the residuals of 12-month YGTSS total tic score (TTS) and the residuals of DCI (**a**), SRS total T (**b**), and CBCL A-A-A (**c**) after regression of baseline TTS. (**d**) Mean TTS at baseline (circle) and at 12-month (triangle) for each categorical predictor.

We tested if clinical features at baseline could predict clinically relevant tic outcome in terms of the 12-month YGTSS TTS. We hypothesized that baseline TTS and PUTS scores would be associated with tic outcome at the 12-month visit. The 12-month TTS was strongly associated with baseline TTS, *r* = 0.456, *p* = 0.004, so additional analyses controlled for baseline TTS using partial correlations and ANCOVAs. PUTS was not associated with tic outcome, *r*_*partial*_ = 0.063, *p* = 0.727 (Spearman). In addition, we examined the candidate predictor variables listed in Tables 3 and 4 based on previous literature (Fig. 1). These additional analyses can be considered exploratory, so the uncorrected p values should be considered with caution.

**Table 3:** Partial correlations between continuous predictor variables and 12-month tic severity (YGTSS total tic score) controlling for baseline tic severity.

Predictor	n	Pearson Correlation		Spearman Correlation	
		partial r	p value	partial r	p value
PUTS total	34	0.094	0.602	**0.063** ^**†**^	**0.727** ^**†**^
DCI total	38	0.327	0.048	**0.320** ^**†**^	**0.054** ^**†**^
ADHD Rating Scale	39	0.103	0.538	**0.089** ^**†**^	**0.594** ^**†**^
CY-BOCS	38	0.12	0.479	**0.195** ^**†**^	**0.246** ^**†**^
Age at tic onset	39	0.264	0.11	**0.192** ^**†**^	**0.248** ^**†**^
Interval_between_visits	39	**−0.202**	**0.225**	−0.193	0.247
K-Bit IQ	39	**−0.13**	**0.438**	−0.05	0.766
SES	39	−0.228	0.169	**−0.174** ^**†**^	**0.297** ^**†**^
SRS total T scores	39	0.482	0.002	**0.312** ^**†**^	**0.056** ^**†**^
- SRS awareness T scores	39	**0.111**	**0.506**	0.079	0.637
- SRS cognition T scores	39	**0.232**	**0.16**	0.251	0.128
- SRS communication T scores	39	0.479	0.002	**0.381** ^**†**^	**0.018** ^**†**^
- SRS motivation T scores	39	0.498	0.001	**0.414** ^**†**^	**0.010** ^**†**^
- SRS mannerisms T scores	39	0.542	0.000	**0.389** ^**†**^	**0.016** ^**†**^
CBCL - A-A-A (DESR)	39	0.362	0.026	**0.406** ^**†**^	**0.011** ^**†**^
- CBCL - Anxious/Depressed	38	0.431	0.008	**0.400** ^**†**^	**0.014** ^**†**^
- CBCL - Attention Problems	39	0.328	0.045	**0.244** ^**†**^	**0.139** ^**†**^
- CBCL - Aggressive Behavior	39	0.242	0.143	**0.268** ^**†**^	**0.103** ^**†**^

Dagger (^**†**^) in the Spearman correlation columns indicates that the data were not normally distributed (tested by Shapiro-Wilk test). CBCL = Child Behavior CheckList; CY-BOCS = Child Yale-Brown Obsessive Compulsive Scale; DCI = Diagnostic Confidence Index; DESR = deficient emotional self-regulation; PUTS = Premonitory Urge for Tics Scale; SES = socioeconomic status; SRS = Social Responsiveness Scale.

**Table 4:** Mean YGTSS total tic score (TTS) at 12 months stratified by categorical variables measured at baseline.

Predictor	Group 1 (N)	mean ± SD	Group 2 (N)	mean ± SD	F	p
Sex	M (26)	14.192 ± 5.685	F (13)	14.077 ± 8.470	0.003	0.956
First-degree relative with tics	Yes (15)	11.933 ± 5.216	No (24)	15.542 ± 7.126	3.445	0.072
- Parent with tics	Yes (9)	12.333 ± 5.220	No (30)	14.700 ± 6.979	1.057	0.311
Phonic tics (ever)	Yes (32)	14.844 ± 6.441	No (7)	11.000 ± 7.047	2.313	0.137
3 or more vocal tics	Yes (11)	18.364 ± 5.853	No (28)	12.500 ± 6.251	8.160	0.007
Tic below the neck	Yes (27)	14.519 ± 6.963	No (12)	13.333 ± 6.005	0.318	0.576
Non-tic K-SADS diagnosis	Yes (31)	14.516 ± 6.708	No (8)	12.750 ± 6.541	0.542	0.466
ADHD diagnosis	Yes (14)	12.786 ± 7.319	No (25)	14.920 ± 6.231	1.159	0.289
OCD diagnosis	Yes (5)	15.600 ± 7.403	No (34)	13.941 ± 6.601	0.334	0.567
Anxiety disorder	Yes (20)	16.000 ± 6.882	No (19)	12.211 ± 5.912	4.250	0.047

F statistic and p value reported for ANCOVA.

One participant was removed from the analysis of DCI total score as an outlier. For the remaining participants, DCI total score trended toward significantly predicting 12-month TTS, controlling for baseline TTS, *r*_*partial*_ = 0.320, *p* = 0.054 (Spearman), such that participants with higher DCI total scores at the baseline visit had higher TTS at the 12-month visit. The estimated Bayes factor BF_10_ was 1.40, indicating weak evidence in favor of the model with DCI total score and baseline TTS over the null model with baseline TTS alone. Partial correlation also revealed a marginally significant relationship between SRS (total) T score at baseline and 12-month TTS, controlling for baseline TTS, *r*_*partial*_ = 0.312, *p* = 0.056 (Spearman). The estimated Bayes factor BF_10_ was 27.48, indicating strong evidence in favor of adding SRS (total) T score to the null model with baseline TTS alone. Correlation analyses were also conducted separately for the five SRS Treatment Subscale T scores (Social Awareness, Social Cognition, Social Communication, Social Motivation, and Autistic Mannerisms). Significant relationships were found between 12-month TTS and Social Communication, Social Motivation, and Autistic Mannerisms Subscales (See Table 3). The CBCL’s Anxious/Depressed, Attention Problems, and Aggressive Behavior syndrome scale T scores were summed to yield a CBCL A-A-A score that is often used to define deficient emotional self-regulation (DESR). Partial correlation analysis revealed a relationship between the CBCL A-A-A scores and 12-month TTS, controlling for baseline TTS, *r*_*partial*_ = 0.406, *p* = 0.011 (Spearman). The estimated Bayes factor BF_10_ was 2.47, indicating weak evidence in favor of adding CBCL A-A-A total score to the null model with baseline TTS alone. Further analysis with each of the subscales revealed a significant relationship with the Anxious/Depressed, but not the Attention Problems or Aggressive Behavior subscores (See Table 3). The CBCL/6-18 was used for all participants. For the scoring of our 5-year-old participants, age was entered as 6 years. The analysis was conducted again without the 5-year-old participants, and CBCL A-A-A scores still significantly predicted the 12-month tic outcome, n = 31, *r*_*partial*_ = 0.452, *p* = 0.012 (Spearman). Other potential continuous predictors, including age at tic onset, the interval between the baseline visit and 12-month follow-up visit, IQ, SES, ADHD Rating Scale, and CY-BOCS, did not significantly predict 12-month TTS controlling for baseline TTS (See Table 3).

For categorical variables, ANCOVA revealed a significant main effect of anxiety disorder (diagnosis of any anxiety disorder at baseline) F(1,37) = 4.25, p = 0.047, as the participants with an anxiety disorder showed more severe 12-month TTS (M = 16.000, SD = 6.882) than the participants who did not have an anxiety disorder (M = 12.211, SD = 5.912). The estimated Bayes factor BF_10_ was 1.51, indicating weak evidence in favor of the model with the presence of anxiety disorder and baseline TTS over the null model with baseline TTS alone. There was a trend toward a significant difference based on family history, such that participants with a first-degree relative(s) with a history of tics (either parents or sibling) showed less severe tic symptoms (M = 11.933, SD = 5.216) than participants without an affected first-degree relative (M = 15.542, SD = 7.126), F(1,37) = 3.445, p = 0.072. As this trend was opposite to the expected direction, the baseline visit TTS was compared between subgroups to test for a possible sample bias. Participants with a first-degree relative with a history of tics had a lower TTS at baseline (n = 15, mean = 16.000, SD = 5.644) than did other participants (n = 24, mean = 17.958, SD = 6.410), but this difference was not statistically significant (independent sample t-test, t(37) = −0.970, p = 0.338). Also, participants with 3 or more phonic tics showed less improvement at follow-up than those with 0–2 phonic tics, F(1,37) = 8.16, p = 0.007. Other potential categorical predictors including sex, phonic tics (ever), and comorbid diagnosis (*i.e*., any non-tic diagnosis on the K-SADS, or ADHD or OCD separately) did not reveal a statistically significant difference (Table 4).

To minimize the effect of multiple comparisons, a stepwise multiple regression analysis was conducted to predict 12-month TTS with all the predictors identified above: baseline TTS, SRS total T score, DCI score, CBCL A-A-A score, and the presence of anxiety disorder. The participant whose DCI value was an outlier (≥3 SD) was excluded from the analysis, so stepwise multiple regression was conducted on the remaining 38 participants. The final model of SRS total T score, baseline TTS, and the presence of anxiety disorder explained nearly half the variance in 12-month TTS (R^2^ = 0.447, F(3,34) = 9.176, p < 0.001; adjusted R^2^ = 0.399). DCI total score and CBCL A-A-A score were excluded from the final model (p > 0.05). See Table 5 for full details on the model at each step.

**Table 5:** Hierarchical multiple regression predicting 12-month TTS from SRS total T score, baseline TTS, and the presence of anxiety disorder.

	12-month TTS					
	Model 1		Model 2		Model 3	
Variable	B	β	B	β	B	β
Constant	−5.546		−11.242		−12.629	
SRS total T score	0.393*	0.486	0.363*	0.449	0.35*	0.433
baseline TTS			0.427*	0.378	0.444*	0.393
Anxiety disorder					3.435*	0.264
R^2^	0.237		0.378		0.447	
F	11.155*		10.635*		9.176*	
ΔR^2^			0.141		0.069	
adjusted R^2^	0.215		0.342		0.399	

N = 38; *p < 0.05; B indicates unstandardized coefficients; β indicates standardized coefficients.

## Discussion

### Outcome

In this, the largest prospective study on the outcome of recent-onset tic disorder, we demonstrated that tics do not remit within a year in most children with recent-onset tics. These results are surprising, given the received wisdom among clinicians that PTD usually is temporary and disappears within a few months^2,10,13–21^, before Tourette syndrome can be diagnosed. In the current study, most children were no longer bothered by their tics one year after tic onset, and several had no tics visible during a long clinical visit that included extensive tic history from parent and child and a neurological exam. However, all children had tics within the past week, sometimes only observed by camera while sitting alone in a separate room. The diagnostic outcome depends in large measure on how remission is defined, as the changes in diagnostic criteria from DSM-IV to DSM-IV-TR to DSM-5 substantially impact the remission rates in these children. According to DSM-IV, which required marked distress or impairment in a life role to diagnose a tic disorder, only 6 of 39 participants met criteria for Tourette’s Disorder at follow-up. However, according to DSM-5, even if all 4 of the participants lost to follow-up remitted completely, at least 90% of the total sample met criteria for Tourette’s Disorder or Persistent (Chronic) Motor Tic Disorder at follow-up (95% CI = 78.4% to 96.3%). Regardless of the diagnostic criteria, we show that tics do not remit in most children within a year, if one includes careful history and adequate direct observation.

Many experts have opined that Provisional Tic Disorder (PTD) usually goes away within a period of months^2,10,13–21^. We note several factors that may explain the apparent disparity. First, in our series, most parents no longer felt the need for clinical attention by the one-year anniversary of their child’s first tic. Therefore, failure to return to the clinic may be misinterpreted as remission of tics. Second, some of the children who had tics when sitting alone showed no evidence of tics during a clinical interview and examination much longer than a typical follow-up visit in a busy clinical practice. Tics tend to be more severe in specific situations, and being alone is one such situation^25^. Thus, the absence of observed tics during a follow-up visit may be misinterpreted as remission of tics. Third, at follow-up, most of the children had no to minimal impairment or distress, and tics without substantial clinical import may not be diagnosed as an illness in routine clinical practice. Fourth, some previous comments on the topic have been muddied by confusion about the definition of transient tic disorder, with many authors confining that term only to those whose tics have already disappeared (discussed in Appendix 1 of Black *et al*.^7^). Naturally prognosis will be better if one focuses only on those whose symptoms have already disappeared at baseline. Finally, the one-year criterion for tic duration required to diagnose a chronic tic disorder is arbitrary, and plausibly full tic remission does occur more commonly, but takes 1.5, 2 or 10 years.

Turning from opinion to data, only 3 previous studies to our knowledge provide direct follow-up in more than a handful of children with PTD, and importantly, our results are broadly consistent with those studies. Bruun and Budman^12^ reported on 58 children with PTD, but much of the follow-up was by phone call, 2–14 years after initial intake. Only 17% of their sample remained remitted over time. Spencer and colleagues retrospectively conducted a structured clinical interview in 36 adults with ADHD who had met criteria for TS or another tic disorder (onset before age 21); tics had remitted in 53% of them after follow-up 1–55 years later^11^. Finally, Shapiro and colleagues followed up by telephone with 26 patients 1–11 years after an initial diagnosis of PTD, and only 35% reported full remission^10^. Overall, the estimate from these prospective studies was that about one third of patients with PTD remitted fully at follow-up periods of a year or more (Appendix 1 of Black *et al*.^7^). Our results suggest that this fraction is even smaller. We highlight that the previous reports had less intensive examination (or none) at follow-up and reported follow-up at substantially longer durations after tic onset than in our study.

The present results also cohere with what is known about tic remission throughout the course of tic disorders over the lifespan. Chronic tic disorders have been reported to remit by late adolescence and early adulthood in roughly one third of patients over 5–10 years of follow-up^26^. However, other studies have shown that tics are still present in 85–90% of TS patients at follow-up, including in subjects who believe their tics have disappeared^27–29^. Furthermore, tics almost always wax and wane in severity while present. As noted by Bruun and Budman^12^, tics often appear with “intervening spontaneous remissions”, and in their experience “complete, life-long remissions are rare”; rather, “the more common course is one of occasional recurrences of mild tics throughout adult life”. Similarly, Shapiro *et al*.^10^ (p.188) reported spontaneous remissions, lasting <1 month to 19 years, in 27.1% of patients with TS, and a recent report highlighted the possible recurrence of tics after decades-long apparent remissions^30^. Thus, full remission is not the typical outcome even when chronic tic disorders appear to remit. Nevertheless, prior to the present report, full remission was expected for most children with PTD.

### Predictors of prognosis

Previous studies suggested variables that might predict future tic outcome, including sex, age, tic phenomenology, tic duration, and comorbidity^7^, and we tested those in this sample. Some significant predictors were features of the tics themselves at baseline. Baseline tic severity (TTS) was significantly correlated with 12-month tic severity (TTS), so we included the baseline TTS in all additional prognostic analyses. Combining DCI score, which measures typical features of TS, with TTS better predicted outcome than did TTS alone, perhaps because DCI reflects the cumulative variety of symptoms whereas YGTSS TTS reflects current severity. Additionally, consistent with the prior report of Shapiro *et al*.^10^ (p.374), three or more phonic tics at baseline predicted worse tic outcome at the follow-up visit.

Interestingly, non-tic symptoms also predicted tic outcome. Higher SRS scores (autism traits), higher CBCL A-A-A scores (deficient emotional self-regulation), and presence of an anxiety disorder at baseline were each associated with worse tic outcome at 12-month follow-up. Together with tics, these may indicate problems with brain systems that are not perfectly tied to our current nosology. For instance, Althoff and colleagues^31^ discussed the A-A-A profile (CBCL-DP) as sensitive to dysregulation in multiple domains, including affect (anxiety/mood), behavior (disruptive), and cognition (attention). They viewed this profile as a global deficit in self-regulation related to processes of effortful control as defined by Eisenberg and Spinard^32^. Mildly elevated SRS scores may similarly indicate dysregulation of communication, social behavior, and motor function. In this view, tics may be another indicator of behavioral dysregulation, with dysregulation in multiple domains indicating greater overall severity, which may predict persistence of tics (and perhaps of other symptoms). More anxious children may also show more tics because tics are exacerbated by moment-to-moment changes in stress or anxiety^33,34^. In the Supplement we discuss alternative potential explanations for the association of these non-tic symptoms at baseline with tic outcome.

### Limitations

The present sample by necessity was not epidemiologically representative; for instance, any study like ours will over-represent parents who notice tics and are able to participate. The typical delay from onset of tics to diagnosis (estimated retrospectively) has been reported as 10 years^35^, highlighting the difficulty in studying the PTD population from clinical samples alone. Understanding that problem, in order to meet the goal of enrolling participants within the first few months after tic onset, we recruited vigorously rather than passively waiting for patients to come to clinical attention. Specifically, we placed advertisements around the community, talked to and left flyers with local physicians, gave information to local elementary schools, and so forth. Therefore, the majority of our sample did not come from the clinic (see Table S1). More concerning is whether the sample was biased to enroll children with features linked to higher or lower remission rate. One example would be positive family history, as discussed above. Another example would be more severely affected children, who may come more quickly to clinical attention. However, the severity at presentation in our sample was not especially high, with TTS generally low at enrollment (mean 17.23 ± 6.15). Additionally, four of the children came from families of pediatric neurologists or child psychiatrists, and nine children had a parent or sibling with tics; both groups would be expected to be sensitive to mild tics. Thus, overall, we contend that the sample is reasonably representative of the population.

### Future directions

We have collected additional data in this sample in other domains, including a standardized tic suppression task^36,37^, a measure of habit learning^38^, the Purdue Pegboard task^39^, structural MRI with automated basal ganglia volumetry^40,41^, and resting state functional connectivity MRI^42^. Those data will be subjects for later reports. Considering the multiple-domains dysregulation hypothesis discussed above, other forms of motor dysregulation may also be relevant (such as poor motor coordination and other subtle neurological signs), so we are assessing more recently enrolled participants with the PANESS^43,44^.

### Conclusions

This study provides for the first time face-to-face assessment and prospective follow-up on a reasonably large sample of children with PTD as soon as practicable after tic onset. Although most children’s tics improve substantially by the first anniversary of their first tic, careful and thorough assessment shows that tics are still present. In addition to greater tic severity and variety at baseline, the presence of an anxiety disorder, high scores on the CBCL A-A-A measure, and higher (though still subsyndromal) SRS scores can provide clinically useful information for predicting higher likelihood that tics will be more severe at follow-up.

## Methods

### Subjects

Participants included were children with current tics whose tics began within the 6 months prior to the baseline study visit. Exclusion criteria included neurological disorder other than tics or migraine, known structural brain disease, mental retardation, DSM-IV-TR autistic disorder, psychosis, mania, current major depression, severe systemic illness, and non-proficiency with the English language. Recruitment sources included clinical referrals (mostly from the Washington University School of Medicine Movement Disorders Center clinic, with some from community doctors or the child neurology and child psychiatry departments) and various advertising methods. Of 105 potential participants contacted, we enrolled 43 (Table 1 for sample characteristics, and Supplementary Table S1 for enrollment by recruitment source).

To determine the best estimate of the date of tic onset, we gathered multiple sources of information, including in-depth discussion about tic onset with the child and parent/guardian at the beginning of the study visit, queries during diagnostic interviews with the child and parent/guardian, and in some cases, reviewing home videos or contacting current and past teachers of the child, as described previously^36^. Repetitive behaviors that were diagnostically ambiguous (e.g., knuckle popping, skin picking) were not considered for dating tic onset. Since the historical information was imperfect, children were not excluded if they had a possible single past tic that had lasted less than 3 months and had disappeared at least one year prior to the current episode. This was the case for three participants (ages 8.5, 12.3 and 14.3 years). Assuming the previous phenomenon was indeed a tic, then these 3 met criteria at baseline for a recurrent episode of Transient Tic Disorder under DSM-IV-TR, but for Tourette’s Disorder under DSM-5. The remaining 40 participants met criteria at screening for both DSM-IV-TR Transient Tic Disorder and DSM-5 PTD (see Table 2).

### Protocol

Children participated in a baseline study visit within 6 months of tic onset. This study visit occurred over two days. The first day consisted of in-depth clinical interviews and assessments, behavioral tasks (including a tic suppression paradigm in which the child was video recorded while sitting alone)^36,37^, and a mock MRI scan. The second day consisted of an MRI scan and, if needed, completion of behavioral tasks. MRI and behavioral task data will be reported separately. Children also participated in a longitudinal follow-up visit on the 12-month anniversary of tic onset, *i.e*., 6–12 months after the baseline study visit. This timing was chosen because accepted diagnostic criteria for a chronic tic disorder require tics to have been present for at least one year. This follow-up visit included clinical assessments in order to assess tic disorder diagnosis and changes in symptoms and comorbidities since the baseline study visit.

### Clinical assessments

At the baseline study visit, psychiatric diagnoses were evaluated using the Kiddie Schedule for Affective Disorders and Schizophrenia (K-SADS)^45^; parents and children were interviewed separately by two of three trained raters who all had masters-level credentials in counseling or social work, at least 3 years’ experience with psychiatric diagnostic interviewing, and additional training with author KJB on psychiatric diagnosis in TS. Symptom severity measures for the past week, including the Yale Global Tic Severity Scale [YGTSS; total tic score range 0–50, impairment score range 0–50^46^], Children’s Yale-Brown Obsessive Compulsive Scale [CY-BOCS; range 0–40^47^], and ADHD Rating Scale [range 0–54^48^], were scored by author KJB after interview, neurological examination, and review of K-SADS data and parent questionnaires. On each of these scales, higher scores indicate greater severity. Typical historical features of TS were assessed by author KJB with the Diagnostic Confidence Index [DCI; range 0–100, higher scores indicate more features of classic TS^33^]. To assess premonitory symptoms, the Premonitory Urge for Tics Scale [PUTS; range 9–36, higher scores indicate more premonitory symptoms^24^] was administered by author KJB to the child with parental help if needed. Author KJB also provided a DSM-5 diagnosis for all children for tic disorders, Attention-Deficit/Hyperactivity Disorder (ADHD), and Obsessive-Compulsive Disorder (OCD) after reviewing all information^49^. The K-BIT II was administered by a trained rater to the child to estimate IQ^50^. Parents completed the following: YGTSS tic symptom checklist, Child Behavior Checklist [CBCL^51^], Social Responsiveness Scale [SRS^52^], Barratt Simplified Measure of Social Status [SES^53^], Pediatric Quality of Life Inventory [PedsQL^54^], and forms to record handedness, history of maternal smoking or other problems during pregnancy, history of birth complications, and pertinent family history. Midway through the study, we added a question to the protocol asking if the parent/guardian would be taking their child to the doctor now or in the near future because of his/her tics.

The follow-up visit included the following: YGTSS, CY-BOCS, ADHD Rating Scale, DCI, PUTS, CBCL, PedsQoL, and clinical examination by author KJB for tic disorder, ADHD, and OCD diagnosis. Study data were managed using REDCap [Research Electronic Data Capture] electronic data capture tools hosted at Washington University^55^.

### Statistical analysis

A major goal of this work was to identify clinical features at baseline that could predict outcome at follow-up. To assess outcome, 12-month YGTSS Total Tic Score was the dependent variable in the analyses. Our original proposal focused on change in YGTSS Total Tic Score over time (ΔTTS) as the dependent variable as a way of simplifying power calculations (http://osf.io/y5vxj), and that analysis appears in the Supplement. However, the ΔTTS is susceptible to a floor effect for participants with low TTS at baseline, and the more clinically relevant outcome is symptom severity at follow up (12-month TTS) rather than degree of improvement.

For the continuous variables, outliers (≥3 SD) were removed from the analysis. Shapiro-Wilk test revealed that most of the variables were not normally distributed, so both Pearson and Spearman partial correlation analyses were conducted between each of the candidate predictor variables and 12-month TTS after controlling for the baseline TTS. In the main text, we reported the results of Pearson partial correlation when the data were normally distributed, and the results of Spearman partial correlation if the data were not normally distributed. For the categorical variables, an ANCOVA was conducted to test if subgroups divided by the candidate predictors showed a different degree of 12-month TTS after controlling for baseline TTS. To minimize the effect of multiple comparisons, a stepwise multiple regression analysis was conducted to predict 12-month TTS with all the predictors that were significantly associated with the tic outcome at 12-month. In addition, to complement the limitation of conventional null hypothesis significant testing^56^, we conducted Bayesian hypothesis testing using JASP^57^ with BIC method. BF_10_ over 3 was considered as positive^58^/substantial^59^ evidence (strong evidence if BF_10_ > 10)^60^.

### Ethics approval and consent to participate

The study was approved by the Washington University Human Research Protection Office (IRB), protocol numbers 201109157 and 201707059. Each child assented and a parent (guardian) gave informed consent prior to study participation. All participants were compensated for their time. All experiments were performed in accordance with relevant guidelines and regulations^61^.

## Supplementary information

Supplemeantary File

Supplementary Dataset 1

## Data Availability

The supplementary data file provides individual participant data.
