# Supplementary material for: Provisional Tic Disorder is not so transient
Source: Sci Rep. 2019 Mar 8;9:3951. doi: 10.1038/s41598-019-40133-4 (PMC6408476; doi:10.1038/s41598-019-40133-4)
Supplement: Supplementary file 1 — Supplemeantary File [file 41598_2019_40133_MOESM1_ESM.docx]

Supplementary information for:

# Provisional Tic Disorder is not so transient

Soyoung Kim^a^

Deanna J. Greene^a,b^

Emily C. Bihun^a^

Jonathan M. Koller^a^

Jacqueline M. Hampton^c^

Haley Acevedo^a^

Angela M. Reiersen^a^

Bradley L. Schlaggar^d,e,f^

Kevin J. Black^a,b,c,g *^

^a^Department of Psychiatry, Washington University School of Medicine, United States

^b^Department of Radiology, Washington University School of Medicine, United States

^c^Department of Neurology, Washington University School of Medicine, United States

^d^Kennedy Krieger Institute, Baltimore, MD, United States

^e^Department of Neurology, Johns Hopkins University School of Medicine, Baltimore, MD, United States

^f^Department of Pediatrics, Johns Hopkins University School of Medicine, Baltimore, MD, United States

^g^Department of Neuroscience, Washington University School of Medicine, United States

# Supplementary Methods

*Table S1. Enrollment by source.*

|  | Clinical | Advertising | Other / unknown |
| --- | --- | --- | --- |
| Contacted | 33 | 31 | 41 |
| Not interested / no show | 9 | 11 | 8 |
| Ineligible | 8 | 10 | 16 |
| Enrolled | 16 | 10 | 17 |

43 participants were recruited in total from Sep/24/2010 through Jun/27/2017.

# Supplementary Results

The identical analysis as reported in prognosis section of the results were conducted with the change in TTS over time (i.e., the 12-month TTS – the baseline TTS, ΔTTS) as dependent variable. As ΔTTS was correlated with baseline TTS (r=−.47, p<.05), baseline TTS was included as a covariate. The correlation analyses revealed the exact same results, so the results are not shown again. The ANCOVA analyses results are shown in Table S2.

Table S2. Mean total tic score (TTS) at 12-month follow-up for each subgroup of the categorical variables and the results of ANCOVA.

| Predictor | Group 1 (n) | mean±SD | Group 2 (n) | mean±SD | F | p |
| --- | --- | --- | --- | --- | --- | --- |
| Sex | M (26) | -2.962 ± 6.63 | F (13) | -3.231 ± 7.002 | 0.017 | 0.897 |
| First relative with tics | Y (15) | -4.067 ± 7.015 | N (24) | -2.417 ± 6.507 | 0.72 | 0.402 |
| Parent with Tics | Y (9) | -1.778 ± 6.22 | N (30) | -3.433 ± 6.847 | 0.517 | 0.477 |
| Vocal tics ever | Y (32) | -3.906 ± 6.65 | N (7) | 0.857 ± 5.581 | 3.553 | 0.068 |
| 3 or more vocal tics | Y (11) | -1.909 ± 6.76 | N (28) | -3.5 ± 6.697 | 0.601 | 0.443 |
| Non-tic K-SADS diagnosis | Y (31) | -3.097 ± 7.162 | N (8) | -2.875 ± 4.612 | 0.009 | 0.927 |
| ADHD diagnosis | Y (14) | -4.786 ± 8.088 | N (25) | -2.08 ± 5.671 | 1.863 | 0.181 |
| OCD diagnosis | Y (5) | 0 ± 7.416 | N (34) | -3.5 ± 6.547 | 1.487 | 0.231 |
| Anxiety disorder | Y (20) | -1.25 ± 7.048 | N (19) | -4.947 ± 5.826 | 4.046 | 0.052 |

# Supplementary Discussion

Previous studies suggested potential variables that might predict future tic outcome, including sex, age, tic phenomenology, tic duration, and comorbidity. We discuss each of these in the following paragraphs.

## Baseline tic severity and tic duration

First of all, we found that tic severity (as measured by the TTS) at baseline was correlated with tic severity at the 12-month follow up. In a previous cross-sectional prevalence study, children with tics for less than a year had lower severity than the children with TS or CTD ^1^. This would predict that the children who had more severe TTS at the baseline visit would show more persistent tics (worsening or less improvement in tics) at the 12-month visit, but our results showed the opposite pattern. The participants who had more severe tics at the baseline visit showed more improvement while the participants who had less severe tics at the baseline visit showed less improvement or worsening of tics. We included the baseline TTS in all the additional prognostic analyses to control baseline differences across the participants.

We also examined whether tic duration at study entry could predict tic outcome at the follow-up visit. At the baseline visit, all the participants had tics less than 6 months, but the duration of tics varied across the participants from 22 to 182 days. Note however that a shorter tic duration at the baseline visit implies a longer interval between the baseline visit and the 12-month visit. We found no relationship between the tic duration at the baseline visit (or the length of the interval between the two visits) and tic outcome at the 12-month follow-up visit.

## Phonic tics and distribution of motor tics

Previous studies suggested that the location or type of tics could predict tic outcome. Shapiro et al.^2^ identified a tic below the neck at the first visit as a factor associated with less favorable outcomes. Similarly, Corbett et al. ^3^ found a trend for fewer remissions in patients with a lower extremity tic at presentation. In the current study, 27 out of 39 participants had tics below the neck at the baseline visit, but the presence of these tics did not predict outcome. Previous studies also reported better prognosis in children who presented without phonic tics ^2,4^. In the current study, 7 out of 39 participants had no history of phonic tics at the baseline visit, but the presence of phonic tics did not predict outcome. Shapiro et al.^2^ also reported that participants with three or more phonic tics during the first year were more likely to develop TS, while all their participants with 0-2 phonic tics remitted. In the current study, tic severity at follow up was worse in children with 3 or more phonic tics at baseline. However, note that all the participants still showed tics at the 12-month follow-up visit regardless of the presence or number of phonic tics at the baseline visit, though most did not meet DSM-III-R (as used in Shapiro et al.^2^ or DSM-IV criteria for TS due to the lack of impairment or marked distress. If the group difference in the number of patients with a diagnosis of TS at the follow-up visit in Shapiro et al.^2^ was due to the presence or absence of marked distress or impairment, then the finding that the patients with three or more phonic tics were more likely to experience marked distress or impairment is not surprising.

## Phenomenology of tics and premonitory symptoms

We examined whether participants who presented with a more classic history of Tourette Syndrome (i.e., higher DCI score) or who reported stronger premonitory urges (i.e., higher PUTS score) at the baseline visit showed worse tic severity at the 12-month follow up visit. DCI scores at baseline significantly predicted tic severity at follow up but PUTS score did not. The relationship between baseline DCI and 12-month TTS might not be surprising given that baseline DCI and baseline TTS were correlated. However, baseline DCI predicted 12-month TTS even after statistically controlling for baseline TTS. While YGTSS assesses the tic symptom severity of the prior week, DCI assesses the lifetime experience of “classic” TS features. Since tics wax and wane, it is possible for an individual to have a low TTS but high DCI. The DCI also includes questions assessing subjective and cognitive experiences (such as suppressibility) that are highly typical of the disorder but not necessarily correlated to high symptom severity ^5^. Our results suggest that future tic outcome can be predicted better considering both TTS and DCI scores rather than TTS alone. Baseline PUTS score, on the other hand, did not predict the 12-month tic outcome. This might be partially because PUTS can be less reliable in children under 10 years old ^6,7^.

## Family History

Khalifa and Von Knorring^1^ reported that 80% of their TS participants had a first-degree relative with a psychiatric disorder. Our study found similar rates in a PTD sample. About 77% of the participants had a first-degree relative with a history of psychiatric disorder (including tics, ADHD or OCD). Specifically, 38% of the participants had a first-degree relative with motor or phonic tics. A first-degree relative with tics might be expected to presage worse outcome, given the heritability of tic disorders ^8^. However, there was no significant difference in tic outcome at the 12-month visit between the participants with or without an affected first-degree relative. It is possible that our sample was biased with regard to family history of tics, as participants whose mother or father had a history of tics tended to show lower TTS at the baseline visit (n=9, mean=14.111, SD=3.516, *vs.* n=30, mean=18.133, SD=6.474, independent sample t-test, t(37)=−1.776, p=.084). This difference may be due to parents with tics themselves being more sensitive to noticing tics in their child.

## SRS Scores

The SRS assesses symptoms related to the autism spectrum, with higher score indicating more autistic traits. In the current study, SRS total T scores at the baseline visit significantly predicted tic severity at the 12-month visit. Note that this study excluded participants with a DSM-IV Autistic Disorder diagnosis, and generally SRS scores in this sample were in the non-clinical range; only 7 children had a score in the mild to moderate range (60 -75 ; Constantino & Gruber, 2005), and none had higher scores. Darrow et al.^10^ reported that the TS population showed higher SRS scores mostly due to the Restricted Interests and Repetitive Behavior (RRB) subscale (originally called Autistic Mannerisms; renamed in SRS-2) ^11^, which needs to be interpreted carefully as it is possible that tic symptoms led to endorsement of items intended to describe non-tic stereotypies. However, we found no significant relationship between current tic severity and SRS total score or the mannerisms subscore at the baseline visit (both p>.05). In fact, exploring the SRS subscales revealed a significant association between tic severity outcome and baseline communication and motivation subscores in addition to mannerisms.

One could imagine that autistic-like symptoms captured by higher SRS scores could exacerbate the distress or social impairment that TS patients experience, rather than tic severity itself. However, in this sample, the YGTSS Impairment score at follow-up was low in almost all participants. Alternatively, children with higher SRS scores may be less sensitive to negative social feedback due to their tics, and hence suppress tics less in social settings, leading to overall higher YGTSS TTS scores at follow-up. Thus, it is possible that these children cannot suppress tics as well as children with lower SRS scores due to less practice over time. However, SRS scores and tic suppression ability were not correlated at the baseline visit (data not shown). Another possibility is that autistic traits (which tend to persist themselves) might generally predict persistence of any co-occurring neurodevelopmental disorders. For example, in a longitudinal study of autistic-like social communication deficits and ADHD symptoms (Avon Longitudinal Study of Parents and Children), children with persistent (rather than childhoood-limited) hyperactive-inattentive symptoms were most likely to show social-communication deficits ^12^. There is also evidence that among children with tics, those with more autistic traits have increased rates of additional neuropsychiatric comorbidities, such as ADHD, rage attacks, and Oppositional-Defiant Disorder ^13^. Considering these studies, the presence of elevated autistic traits may indicate the presence of a more severe, complex, and persistent disorder.

## CBCL Scores

The CBCL assesses a range of behavioral and emotional problems in children, and provides eight different Syndrome Scales ^14^. Previous studies utilized the sum of three of these scales - Anxious/Depressed, Attention Problems, and Aggressive Behavior (A-A-A scale) - to categorically define deficient emotional self-regulation (DESR; i.e., emotional lability, as a score of 180 to 210 ^15,16^) and severe dysregulation with a presumed risk of developing bipolar disorder (score ≥ 210 ^17^). Althoff and colleagues describe the same CBCL profile as the CBCL Dysregulation Profile (CBCL-DP) and suggest it measures a broad range of dysregulation, including affective, behavioral, and cognitive domains ^18^. Emotional dysregulation has been reported as common in TS patients ^19^, and a study that assessed the CBCL A-A-A scale in adolescents with TS found evidence for emotional lability unrelated to comorbidity ^20^. Interestingly, 60% of those TS participants showed CBCL A-A-A scores ≥ 180 and 15% of them showed scores > 210. In our sample, 6 out of 43 (14%) participants scored 180 to 210 and no participant scored over 210 at the baseline visit. Nevertheless, CBCL A-A-A scale scores at screening predicted tic severity at follow up. While prognostic significance of the CBCL A-A-A scale has been reported in children with ADHD ^21^, a similar predictive value has not previously been demonstrated in a tic disorder population.

## ADHD, OCD and Anxiety

The majority of the TS population has one or more psychiatric comorbidity such as ADHD, OCD, or anxiety disorder ^22^. We carefully examined our participants and identified participants with ADHD, OCD or anxiety disorder at baseline. We tested whether their tic outcome at 12 months differed depending on comorbidity. Perhaps surprisingly, ADHD or OCD did not significantly predict outcome. ADHD and OCD are the most common comorbid disorders in TS ^23^, and have been suggested to predict future tic outcomes in chronic tic disorders. Spencer et al.^24^ conducted a prospective study and found that boys with ADHD showed 5 times more tic disorders at baseline. Furthermore, those who did not have tics at baseline were 7 times more likely to have new onset tics by 4-year follow-up compared with a control group without ADHD. The high prevalence and incidence of tic disorder in boys with ADHD suggests (though does not prove) that ADHD might predict longer persistence of tic disorder.  Peterson et al.^25^ showed that ADHD symptoms at early adolescence could predict tic persistence into late adolescence, and OCD symptoms in late adolescence could predict tic persistence into early adulthood. In the current study, the participants diagnosed with ADHD or OCD at the baseline visit did not show different tic outcome at the 12-month visit. Neither did symptom severity at the baseline visit, using the ADHD rating scale or CY-BOCS. The different results might be due to the difference in age between studies. Our participants were younger than the age range that was suggested as a critical period in which certain comorbid disorders could predict future tic persistence. Previous studies have suggested that tic disorder, ADHD, and OCD are tightly related, so it is plausible for one disorder to predict the course of the other disorder. However, these syndromes are known to have different developmental time courses, such that on average, the onset of ADHD symptoms precedes the onset of tics by 2-3 years Kumar, Trescher, & Byler, (2016), while the period of worst-ever OCD symptoms follows the peak of tic symptoms by about 2 years ^27^. Unlike ADHD and OCD, we found that the participants with an anxiety disorder (other than OCD) at the baseline visit showed significantly higher TTS at the 12-month visit. The individuals who had any anxiety disorder at the baseline visit showed more severe TTS at the 12-month visit than the rest of the participants. Consistent results were also found in that the Anxious/Depressed CBCL subscale scores at the baseline visit could predict the tic outcome at the 12-month visit. Why might anxiety predict clinical tic outcome? Possible explanations include the following. (1) Anxiety is conceptualized as being sustained by negative reinforcement, so perhaps anxious children are “better” at negative reinforcement learning. Under the traditional model for habit reversal therapy for tic disorders, this would tend to lead to more persistent tics. However, several recent empirical studies cast doubts on the idea that behavior therapy benefits tics by interrupting negative reinforcement ^28^. (2) Children with anxiety may be more likely to endorse cognitive distortions about tics such as “If I repress my tics, I’ll blow up,” one of the items captured by the Beliefs About Tics Scale (BATS^29^). Such beliefs would tend to undermine efforts to suppress tics. We have no BATS data in this sample. (3) Tics are exacerbated by moment-to-moment changes in stress or anxiety ^5,30^, and children with higher trait anxiety may thus show more tics throughout the week. These 3 ideas may also help explain the link with the A-A-A score, as the anxiety symptom scale was the A-A-A component linked most strongly to outcome.

## Other

Age of onset has been previously identified as a possible predictor of tic outcome. For example, Corbett et al.^3^ found that complete remission was more likely if tics began between 6 and 8 years than between 2-5 years or 9-10 years. However, in the current study there was no significant relationship between the age of tic onset and tic symptom outcome (not even a non-linear relationship, data not shown). Another potentially predictive variable is sex, yet previous findings are mixed. One study reported that girls with a tic disorder were more likely to show spontaneous remission than boys ^31^, while a similar study found no relationship between remission and sex ^32^. In the current study, we found no significant difference between male and female participants in tic outcome at the 12-month visit. In addition, neither K-Bit IQ nor SES was related to tic outcome at 12-month visit. These findings suggest that standard demographic information may not be reliable predictors of tic outcomes.

##

# Supplementary References

1. Khalifa, N. & Von Knorring, A. L. Tourette syndrome and other tic disorders in a total population of children: Clinical assessment and background. *Acta Paediatr. Int. J. Paediatr.* **94,** 1608–1614 (2005).

2. Shapiro, A. K., Shapiro, E. S., Young, J. G. & Feinberg, T. E. *Gilles de la Tourette Syndrome*. (Raven Press, Publishers, 1988).

3. Corbett, J. A., Mathews, A. M., Connell, P. H. & Shapiro, D. A. Tics and Gilles de la Tourette’s syndrome: A follow-up study and critical review. *Br. J. Psychiatry* **115,** 1229–1241 (1969).

4. Bruun, R. D. & Budman, C. L. The course and prognosis of Tourette syndrome. *Neurol. Clin.* **15,** 291–298 (1997).

5. Robertson, M. M. *et al.* The Tourette syndrome diagnostic confidence index. *Neurology* **53,** 2108–2112 (1999).

6. Steinberg, T. *et al.* Tic disorders and the premonitory urge. *J. Neural Transm.* **117,** 277–284 (2010).

7. Woods, D. W., Piacentini, J., Himle, M. B. & Chang, S. Premonitory Urge for Tics Scale (PUTS): initial psychometric results and examination of the premonitory urge phenomenon in youths with Tic disorders. *J. Dev. Behav. Pediatr.* **26,** 397–403 (2005).

8. Hyde, T. M., Aaronson, B. A., Randolph, C., Rickler, K. C. & Weinberger, D. R. Relationship of birth weight to the phenotypic expression of Gilles de la Tourette’s syndrome in monozygotic twins. *Neurology* **42,** (1992).

9. Constantino, J. N. & Gruber, C. *Social Responsiveness Scale (SRS) Manual*. (Western Psychological Services, 2005).

10. Darrow, S. M. *et al.* Autism Spectrum Symptoms in a Tourette’s Disorder Sample. *J. Am. Acad. Child Adolesc. Psychiatry* (2017). doi:10.1016/j.jaac.2017.05.002

11. Constantino, J. N. & Gruber, C. P. *Social responsiveness scale (SRS-2)*. (Western Psychological Services, 2012).

12. St. Pourcain, B. *et al.* Links between co-occurring social-communication and hyperactive-inattentive trait trajectories. *J. Am. Acad. Child Adolesc. Psychiatry* **50,** 892–902.e5 (2011).

13. Pringsheim, T. & Hammer, T. Social behavior and comorbidity in children with tics. *Pediatr. Neurol.* **49,** 406–410 (2013).

14. Achenbach, T. M. & Rescorla, L. A. *Manual for ASEBA school-age forms and profiles*. *Research Centre for Children, Youth and Families, …* (University of Vermont, Research Center for Children, Youth, & Families, 2001).

15. Spencer, T. J. *et al.* Toward defining deficient emotional self-regulation in children with attention-deficit/hyperactivity disorder using the Child Behavior Checklist: a controlled study. *Postgrad. Med.* **123,** 50–59 (2011).

16. Biederman, J. *et al.* Severity of the aggression/anxiety-depression/attention child behavior checklist profile discriminates between different levels of deficits in emotional regulation in youth with attention-deficit hyperactivity disorder. *J. Dev. Behav. Pediatr.* **33,** 236–243 (2012).

17. Biederman, J. *et al.* The child behavior checklist-pediatric bipolar disorder profile predicts a subsequent diagnosis of bipolar disorder and associated impairments in ADHD youth growing up: A longitudinal analysis. *J. Clin. Psychiatry* **70,** 732–740 (2009).

18. Althoff, R. R., Verhulst, F. C., Rettew, D. C., Hudziak, J. J. & Van Der Ende, J. Adult outcomes of childhood dysregulation: A 14-year follow-up study. *J. Am. Acad. Child Adolesc. Psychiatry* **49,** 1105–1116.e1 (2010).

19. Ludolph, A. G., Roessner, V., Münchau, A. & Müller-Vahl, K. Tourette Syndrome and Other Tic Disorders in Childhood, Adolescence and Adulthood. *Dtsch. Aerzteblatt Online* (2012). doi:10.3238/arztebl.2012.0821

20. Rizzo, R., Gulisano, M., Pellico, A., Calì, P. V. & Curatolo, P. Tourette syndrome and comorbid conditions: A spectrum of different severities and complexities. *J. Child Neurol.* **29,** (2014).

21. Biederman, J. *et al.* Longitudinal course of deficient emotional self-regulation CBCL profile in youth with ADHD: prospective controlled study. *Neuropsychiatr. Dis. Treat.* **8,** 267–276 (2012).

22. Hirschtritt, M. E. *et al.* Lifetime prevalence, age of risk, and genetic relationships of comorbid psychiatric disorders in tourette syndrome. *JAMA Psychiatry* **72,** 325–333 (2015).

23. Freeman, R. D. *et al.* Tic disorders and ADHD: Answers from a world-wide clinical dataset on Tourette syndrome. *Eur. Child Adolesc. Psychiatry* (2007). doi:10.1007/s00787-007-1003-7

24. Spencer, T. J. *et al.* The 4-year course of tic disorders in boys with attention- deficit/hyperactivity disorder. *Arch. Gen. Psychiatry* **56,** 842–847 (1999).

25. Peterson, B. S., Pine, D. S., Cohen, P. & Brook, J. S. Prospective, longitudinal study of tic, obsessive-compulsive, and attention-deficit/hyperactivity disorders in an epidemiological sample. *J. Am. Acad. Child Adolesc. Psychiatry* **40,** 685–695 (2001).

26. Kumar, A., Trescher, W. & Byler, D. Tourette Syndrome and Comorbid Neuropsychiatric Conditions. *Curr. Dev. Disord. Reports* **3,** 217–221 (2016).

27. Bloch, M. H. *et al.* Adulthood outcome of tic and obsessive-compulsive symptom severity in children with Tourette syndrome. *Arch. Pediatr. Adolesc. Med.* (2006). doi:10.1001/archpedi.160.1.65

28. Houghton, D. C. *et al.* Investigating Habituation to Premonitory Urges in Behavior Therapy for Tic Disorders. *Behav. Ther.* **48,** 834–846 (2017).

29. Steinberg, T. *et al.* Tic-related cognition, sensory phenomena, and anxiety in children and adolescents with Tourette syndrome. *Compr. Psychiatry* **54,** 462–466 (2013).

30. Conelea, C. A. & Woods, D. W. The influence of contextual factors on tic expression in Tourette’s syndrome: A review. *J. Psychosom. Res.* **65,** 487–496 (2008).

31. Zausmer, D. M. & Dewey, M. E. Tics and heredity. A study of the relatives of child tiqueurs. *Br. J. Psychiatry* **150,** 628–634 (1987).

32. Torup, E. A Follow-Up Study of Children with Tics. *Acta Paediatr.* **51,** 261–268 (1962).
